# Impact of Selection Signature on Genomic Prediction and Heritability Estimation in Livestock

**DOI:** 10.3390/ani15101383

**Published:** 2025-05-10

**Authors:** Hongzhi Zhang, Zhixu Pang, Wannian Wang, Liying Qiao, Wenzhong Liu

**Affiliations:** 1 Department of Animal Genetics, Breeding and Reproduction, College of Animal Science, Shanxi Agricultural University, Taigu 030801, China; zhanghongzhi336@163.com (H.Z.); pang_z_x@163.com (Z.P.); wannian1876@163.com (W.W.); liyingqiao1970@163.com (L.Q.); 2 Key Laboratory of Farm Animal Genetic Resources Exploration and Precision Breeding of Shanxi Province, Taigu 030801, China

**Keywords:** genomic selection, heritability estimation unbiasedness, MAF–effect size relationship, GBLUP, MAF stratification

## Abstract

Natural or artificial selection could shape genetic architecture, e.g., the relationship between minor allele frequency (MAF) and the effect sizes of causal variants (CVs). This study aimed to investigate the impact of the MAF–effect size relationship (as a selection signature, *S*) on genomic prediction and heritability estimation in livestock, using both simulated data (Holstein) and real datasets (Holstein and pigs). We evaluated the performance of two models: (1) selection-adjusted genomic best linear unbiased prediction (GBLUP-S), and (2) MAF-stratified selection-adjusted genomic best linear unbiased prediction (GBLUP-SMS). Simulation results demonstrated that for traits under strong negative selection (*S* < −1), both GBLUP-S and GBLUP-SMS outperformed classic GBLUP. The prediction accuracy of GBLUP-S improved by 0.011–0.031, while GBLUP-SMS achieved a gain of 0.005–0.025. Furthermore, GBLUP-SMS exhibited lower sensitivity to variations in *S-*values, whereas GBLUP-S heavily relied on accurate *S* specification. When the true *S* was matched, GBLUP-SMS generated more unbiased (or comparable) heritability estimates and higher prediction accuracy relative to GBLUP-S. Critically, mismatched *S* in GBLUP-S led to increased bias in heritability estimates and reduced prediction accuracy. Cross-validation with real phenotypic data from Holsteins and pigs demonstrated that implementing selection-adjust methods improved prediction accuracy by 0.015 for FP in Holsteins and 0.01 for T1 in pigs, while enhancing the unbiasedness of heritability estimates across all traits. Negative selection signatures were identified for cattle (*S* = −0.5) and pig T1, T2, and T3 (*S* = −1.5, −1, and −2, respectively). These findings advance the theoretical framework of GBLUP-based genomic prediction and heritability estimation.

## Simple Summary

Genomic best linear unbiased prediction (GBLUP) has become a widely adopted method for animal genomic evaluation. This method directly estimates genomic estimated breeding value (GEBV) by constructing a genomic relationship matrix (GRM). The GRM is central to GBLUP and remains an active research topic, as different GRM construction methods correspond to different assumptions. In this study, two selection-adjusted GRMs were compared. Both models were evaluated for their accuracy in GEBV prediction and their performance in heritability estimation under varying selection signatures. The results demonstrated that explicitly modeling the relationship between marker effect sizes and minor allele frequency (MAF) during GRM construction can effectively improve the accuracy of GEBV predictions and enhance heritability estimation performance.

## 1. Introduction

Genomic selection (GS) has been widely applied in livestock and plant breeding programs [1,2]. The central task of GS is the prediction of genomic estimated breeding value (GEBV). Since the first statistical method for GS was proposed by Meuwissen et al. [3], new methods have continually emerged [4,5]. Under the linear model framework, there are mainly two types of methods: derivatives of best linear unbiased prediction (BLUP) [6] and Bayesian Alphabet methods [7,8]. The former methods calculate GEBV by constructing the genomic relationship matrix (GRM) [9], exemplified by genomic best linear unbiased prediction (GBLUP) [10], single-step genomic best linear unbiased prediction (SSGBLUP), and their extensions [11], while the latter methods indirectly derive GEBVs through marker effect estimation, such as BayesA [3]. However, no consensus exists on the optimal GS method for the prediction of specific traits.

In human genetic studies, natural selection has been shown to shape genetic architecture, e.g., the relationship between minor allele frequency (MAF) and the effect sizes of causal variants (CVs) [12]. Negative selection can cause a negative MAF–effect size correlation of CVs (i.e., lower MAF, bigger effect size), whereas positive selection reverses this trend. The linkage-disequilibrium-adjusted kinship (LDAK) model, designed for single nucleotide polymorphism (SNP)-based heritability estimation, accounts for the effect caused by selection through integrating a scale parameter α that captures the relationship between MAF and the effect sizes of SNPs in the GRM [13]. When linkage disequilibrium (LD) and genotype certainty are ignored, the GRM under strong negative selection assumption (i.e., α = −1) matches that of the genome-wide complex trait analysis (GCTA) model [14]; whereas the GRM under neutrality assumption (i.e., α = 0) aligns with the method in [10], which is commonly used in animal and plant prediction. Although the impact of selection on heritability estimation has been widely studied in human diseases [15], its implications for livestock heritability estimation and genomic prediction remain poorly understood.

Classic GBLUP uses a single GRM to estimate the similarity between individuals, implying that the effect sizes of all SNPs follow a common Gaussian distribution [16], which is often unrealistic. To address this, Speed proposed MultiBLUP, which constructs GRMs by SNP classification [17]. Additionally, a series of methods have been proposed for the heritability estimation by stratifying SNPs based on MAF and LD [18], such as genomic restricted maximum likelihood (GREML)-LDMS [19]. While the effectiveness of LD-stratified GRMs has been explored for GS [20], the suitability of MAF-stratified GRMs for GS remains to be investigated.

The objectives of this study were as follows: (1) validate selection-adjusted GRM strategies for genomic prediction in related livestock using Holstein simulations; (2) compare model performance under different selection signatures (MAF–effect size relationships) and GRM approaches (single vs. multi-GRM) in Holstein and pig datasets; (3) quantify trait-specific selection signatures in both species. These results may provide innovative perspectives for optimizing the implementation of genomic selection (GS) in livestock breeding programs.

## 2. Materials and Methods

### 2.1. Population and Genotypes

#### 2.1.1. Holstein

This study utilized publicly available genomic data from 5024 German Holstein dairy cattle generated using the Illumina BovineSNP50 BeadChip (Illumina, Inc., San Diego, CA, USA) [21]. Quality control (QC) for SNPs was performed using PLINK 1.90 based on the following: (1) SNPs with missing call rates > 0.05 were removed; (2) SNPs deviating significantly from Hardy–Weinberg equilibrium (HWE) *p*-value < 10 × 10^−6^ were excluded; (3) SNPs with MAF < 0.01 were discarded. After QC, 42,214 SNPs were retained for subsequent analyses.

#### 2.1.2. Pig

For pig data, this study employed publicly available Porcine SNP60 BeadChip (Illumina, Inc., San Diego, CA, USA) data from the Pig Improvement Company (PIC) (Oxfordshire, UK) [22], comprising 3534 individuals. SNPs located on the X and Y chromosomes were excluded. The overall genotype missing rate across the dataset was less than 0.01. Missing genotypes were imputed by Hickey et al. [22] using parental genotype information, resulting in imputed genotypes represented as decimal values. To ensure compatibility with downstream analytical software, these imputed genotypes were rounded to the nearest integer (0, 1 or 2). QC procedures identical to those described for Holstein were applied, retaining 50,435 SNPs after filtering.

### 2.2. Phenotypes

#### 2.2.1. Simulated Phenotypes

Using QC-filtered SNPs from Holstein, 1000 SNPs were randomly selected as CVs. To simulate the relationship between MAF and the marker effect sizes of CVs, the effect size of each CV was calculated as $g_{i}=\sqrt{[{2p}_{i}\left(1-p_{i}\right){]}^{S}({g_{i}}^{*}{)}^{2}}$, where ${g_{i}}^{*}\;\sim \;N(0,\;1)$ and *p_i_* represents the MAF of the *i*-th CV. Seven *S-*values (−2, −1.5, −1, −0.5, 0, 0.5, and 1) were used to simulate traits under varying selection signatures. Residuals were sampled from the distribution *e_i_* ~ $N\left(0,{\;\sigma }_{a}^{2}(1\;-h^{2})/h^{2})\right)$, where $\sigma_{a}^{2}$ is the total genetic variance, and *h*^2^ is the heritability (fixed at 0.1). The phenotype *y_j_* for individual *j* was derived as $y_{j}=\sum_{i=1}^{m}x_{ij}g_{j}+e_{i}$, where *x_i_* is the genotype (coded as 0, 1, or 2) of individual *j* at the *i*-th CV. The simulation was repeated 10 times to minimize sampling variability and was implemented in R.

#### 2.2.2. Real Holstein Traits

Three economically important traits were analyzed: milk yield (MY), milk fat percentage (FP), and somatic cell score (SCS). Estimated breeding values (EBVs) for these traits were derived using traditional methods and used as pseudo-phenotypes. These traits represent distinct genetic architectures: FP is influenced by a major-effect gene and numerous minor-effect genes, MY is influenced by a few moderate-effect genes and minor-effect genes, and SCS is influenced solely by minor-effect genes [23,24]. Prior to heritability estimation and genomic prediction, pseudo-phenotypes were adjusted for population means and fixed effects, resulting in zero-mean value. Standardization was further applied to ensure a standard deviation of 1 for each trait.

#### 2.2.3. Real Pig Traits

Data for three traits (T1, T2, and T3) were analyzed. Environmental effects (birth year and farm) were corrected for all traits. Trait T3 was centered to a zero mean. For traits T1 and T2, pseudo-phenotypes were calculated for individuals without direct phenotypes using weighted progeny information. Single-trait best linear unbiased prediction (BLUP) was employed to estimate EBVs, which were then used as pseudo-phenotypes in this study.

### 2.3. Models for Genomic Selection and Heritability Estimation

This study implemented two enhanced GBLUP models for genomic prediction and heritability estimation: (1) the selection-adjusted GBLUP (GBLUP-S) model, referred to as the “α model” in [12] and applied to heritability estimation in human traits [25]; and (2) the MAF-stratified selection-adjusted GBLUP (GBLUP-SMS) model, an extension of MultiBLUP [17].

#### 2.3.1. GBLUP-S Model

We used a mixed linear model:

(1) y=Za+e

where ***y*** is a vector of phenotypes corrected for fixed effect and overall mean, ***a*** is a vector of additive genetic values with ***a*** ~ N(**0**, ***G***$\sigma_{a}^{2}$), ***G*** is the GRM, $\sigma_{a}^{2}$ is additive genetic variance, ***Z*** is an incidence matrix corresponding to ***a***, and ***e*** is a vector of residuals with each element ~$N\left(0,\;\sigma_{e}^{2}\right)$, with $\sigma_{e}^{2}$ as the residual variance.

The GRM was constructed as follows:

(2) $$G=\frac{MM^{\prime }}{\sum_{i=1}^{m}{\left[2p_{i}\left(1-p_{i}\right)\right]}^{S+1}}$$

where ***M*** is a centered genotype matrix with each element $m_{\mathrm{ij}}=\left(x_{\mathrm{ij}}\;-\;2p_{i}\right)\;\times \;{\left[2p_{i}\left(1\;-\;p_{i}\right)\right]}^{\frac{S}{2}}$, *x_ij_* is the genotype (coded as 0, 1, or 2) of individual *j* at the *i*-th SNP, and *S* is the assumed MAF–effect size relationship.

#### 2.3.2. GBLUP-SMS Model

In this model, the SNPs were partitioned into five groups by MAF bin (0.01–0.1, 0.1–0.2, 0.2–0.3, 0.3–0.4, and 0.4–0.5).

We used a multi-component mixed linear model:

(3) $$y=\sum_{i=1}^{5}Z_{t}a_{t}+e$$

where ***y*** and ***e*** are the same as for Equation (1), ***a_t_*** is a vector of genetic values of individuals captured by SNPs in the *t*-th groups, with ***a_t_*** ~ N(**0**, ***G***^(*t*)^ $\sigma_{a\;}^{2}$^(*t*)^), *G*^(*t*)^ as the GRM constructed using SNPs in the *t*-th groups, following the same method as described in Equation (2), $\sigma_{a\;}^{2}$^(*t*)^ is the additive genetic variance for the *t*-th bin, and ***Z_t_*** is the incidence matrix for ***a_t_***.

### 2.4. Model Assessment

The genomic prediction and heritability estimation in this study were performed using LDAK 5.2 software [26], while data analysis and visualization were conducted using the R programming (v. 4.5.0).

For simulated data, each scenario was replicated 10 times. The reference and candidate populations were split in a 9:1 ratio. The performance of genomic prediction was assessed using the Pearson correlation coefficient between true breeding values (TBVs) and the GEBVs of individuals in the candidate population. Additionally, the log-likelihood ratio test statistic (LRT) was used to evaluate the goodness of model fit.

For real data, a 10-fold cross-validation approach was employed. Genomic prediction performance was assessed using the Pearson correlation coefficient between phenotypes and GEBVs. Heritability enrichment (defined as the ratio of observed heritability to expected heritability) facilitated understanding of the genetic architecture of complex traits [27]. Heritability enrichment served as an indicator to evaluate the performance of heritability estimation under different parameters of *S* in the GBLUP-SMS model. Genotypic data were stratified into five MAF bins to construct GRMs. The heritability enrichment for the *i*-th MAF bin was calculated as follows:

(4) $$Enrichment_{i}=\frac{{\hat{h}}_{SNP\in MAF_{i}}^{2}}{E({\hat{h}}_{SNP\in MAF_{i}}^{2})}$$

where ${\hat{h}}_{\mathrm{SNP}\in \mathrm{MA}F_{i}}^{2}$ is the estimated SNP heritability explained by the *i*-th bin, and $E({\hat{h}}_{SNP\in MAF_{i}}^{2})$ is the expected SNP heritability explained by the *i*-th bin, computed as follows:

(5) $$E({\hat{h}}_{SNP\in MAF_{i}}^{2})=\frac{\sum_{j\in MAF_{i}}{\left[2p_{j}(1-p_{j})\right]}^{S+1}}{\sum_{i=1}^{5}\sum_{j\in MAF_{i}}{\left[2p_{j}(1-p_{j})\right]}^{S+1}}$$

Heritability enrichment proportions close to 1:1:1:1:1 across the five bins indicates superior model fit and reliable heritability estimates. A chi-square statistic was constructed to evaluate the goodness-of-fit of the G-SeMS model under a different *S*:

(6) $$\chi^{2}=\sum_{i=1}^{5}\frac{{\left(O_{i}-E_{i}\right)}^{2}}{E_{i}}$$

where *O*_i_ is the observed heritability enrichment proportion of the *i*-th bin and *E*_i_ is the expected heritability enrichment proportion of the *i*-th bin. Five MAF bins were tested to assess whether heritability enrichment proportions followed a uniform distribution.

## 3. Results

### 3.1. Genetic Architecture for Simulated Traits

**Figure 1:**
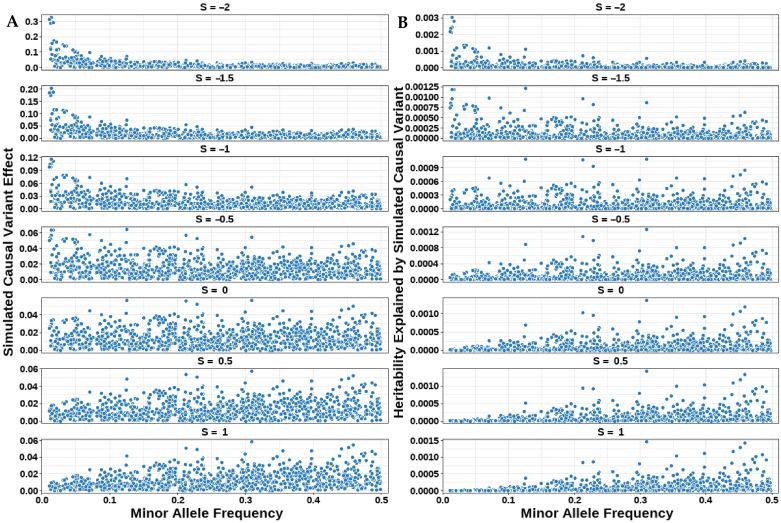
Genetic architecture of simulated traits across seven S-values. (**A**) Distribution of simulated effect sizes from CVs. (**B**) Distribution of heritability explained by simulated CVs.

This study simulated multiple traits with heritability 0.1 across seven *S*-values using real genotypes of Holstein to assess the performance of genomic prediction and heritability estimation for GBLUP-S and GBLUP-SMS models. Figure 1 illustrates the genetic architecture of these simulated traits.

The relationship between MAF and the effect sizes of CVs is shown in Figure 1A. The figure demonstrates that when the *S* = −2, effect sizes were higher in low-MAF regions and lower in high-MAF regions, displaying a left-skewed distribution pattern indicative of strong negative selection. As the *S*-value increased, effect sizes gradually decreased in low-MAF regions and increased in high-MAF regions. At *S* = 0, effect sizes were uniformly distributed across MAF intervals. When *S* = 1, higher marker effect sizes were observed in high-MAF regions, resulting in a right-skewed distribution.

The relationship between heritability and the MAF of CVs under different *S*-values is illustrated in Figure 1B. The heritability explained by the *i*-th CV was calculated as $h_{i}^{2}=2p_{i}(1\;-p_{i})\sigma_{g_{i}}^{2}$, where the simulated variance followed $\sigma_{g_{i}}^{2}\propto [2p_{i}(1\;-p_{i}){]}^{S}$. When *S* = −1, $E(h_{i}^{2})\propto 1$, corresponding to a uniform distribution of SNP heritability across MAF intervals as shown in the figure. For *S* < −1, the heritability distribution exhibited a “left-skewed” pattern, while for *S* > −1, it displayed a “right-skewed” distribution.

### 3.2. Performance of Different Models in Terms of Generic Evaluation and Heritability Estimation in Simulated Data

**Figure 2:**
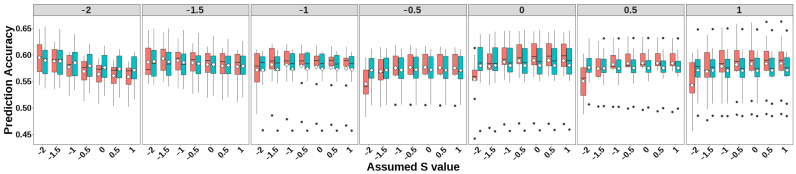
Accuracy of genomic prediction of GBLUP-S (red) and GBLUP-SMS (cerulean blue) at different assumed *S*-values. The white diamonds inside the boxes represent the mean values. The numbers at the top of each plot indicate the true *S*-values used in simulating the traits, while the values on the *x*-axis correspond to the *S*-values applied in the model.

**Figure 3:**
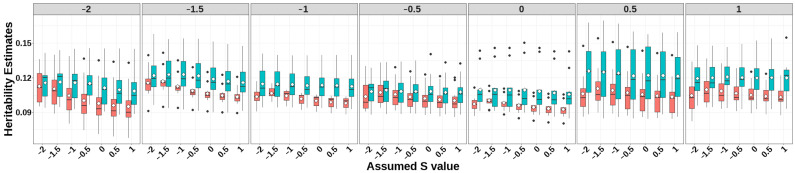
Heritability estimates of GBLUP-S (red) and GBLUP-SMS (cerulean blue) at different assumed *S*-values. The white diamonds inside the boxes represent the mean values. The numbers at the top of each plot indicate the true *S*-values used in simulating the traits, while the values on the *x*-axis correspond to the *S*-values applied in the model.

**Figure 4:**
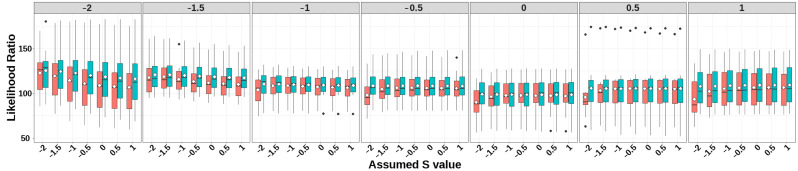
LRT statistics of GBLUP-S (red) and GBLUP-SMS (cerulean blue) at different assumed *S*-values. The white diamonds inside the boxes represent the mean values. The numbers at the top of each plot indicate the true *S*-values used in simulating the traits, while the values on the *x*-axis correspond to the *S*-values applied in the model.

The prediction accuracies of GEBV, heritability estimates, and LRT for simulated traits with heritability 0.1 under different selection signatures are presented in Figure 2, Figure 3, and Figure 4, respectively. Detailed numerical values of genomic prediction accuracies and heritability estimates are provided in Table S1.

Both GBLUP-S and GBLUP-SMS methods demonstrated higher prediction accuracies when the *S* parameters approximated the true selection signature. Notably, GBLUP-S exhibited greater sensitivity to assumed *S*-value specification compared to GBLUP-SMS, with the latter showing smaller accuracy fluctuations across different simulated selection signatures. For simulated traits with selection signatures ≥ 0, GBLUP-S outperformed GBLUP-SMS across all *S*-values except at −2 and −1.5. Compared to the classic GBLUP method (as proposed by Vanranden et al., equivalent to GBLUP-S with *S* = 0), both methods showed great advantages when applied to traits under strong negative selection (selection signatures < −1) using *S*-values close to the true selection signatures: GBLUP-S achieved accuracy improvements of 0.011–0.031, while GBLUP-SMS showed gains of 0.005–0.025.

For all simulated traits with varying selection signatures, GBLUP-SMS consistently produced higher heritability estimates with greater stability compared to GBLUP-S (Figure 3; Table S1). The GBLUP-S method achieved elevated heritability estimates when the specified *S* parameters approximated the true selection signature of the simulated trait. Compared to the classic GBLUP, GBLUP-S generally generated higher heritability estimates when appropriate *S*-values were applied. Notably, both methods exhibited relatively large values in heritability estimates compared to the true heritability (0.1). Notably, no significant concordance was observed between GEBV prediction accuracies and heritability estimates using the two models.

As evidenced by the LRT statistics, while discernible differences in LRT statistics emerged across varying selection signature (S) parameters in the two models, both methods demonstrated adequate model fit. This indicates that the derived heritability estimates maintained satisfactory unbiasedness. Furthermore, a notable consistency emerged between the magnitude of LRT statistics and the heritability estimates, particularly for simulated traits under strong negative selection (S < −1).

### 3.3. Application to Real Traits

#### 3.3.1. Holstein

**Figure 5:**
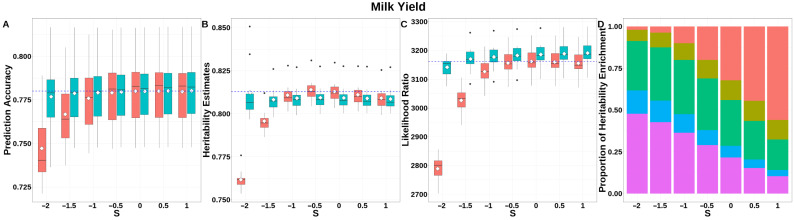
Performance of GBLUP-S (red) and GBLUP-SMS (cerulean blue) at different assumed *S*-values for milk yield in Holstein. (**A**) Genomic prediction, (**B**) heritability estimation, (**C**) LRT statistics, and (**D**) heritability enrichment propositions across MAF bins obtained using GBLUP-SMS. In (**A**–**C**), white diamonds indicate mean values; blue dashed lines represent classic GBLUP. In D, red, olive yellow, dark green, blue, and purple correspond to MAF bins 0.01–0.1, 0.1–0.2, 0.2–0.3, 0.3–0.4, and 0.4–0.5, respectively.

For MY (Figure 5, Table S2), GEBV prediction accuracies for both GBLUP-S and GBLUP-SMS stabilized with negligible variation at *S* ≥ −0.5. GBLUP-SMS maintained robust accuracy across all *S*-values, while GBLUP-S exhibited lower overall performance. The integrated analysis of LRT statistics, MAF-stratified enrichment ratios across varying *S*-values in the GBLUP-SMS model, and their corresponding chi-square statistics (Table S4) demonstrated that GBLUP-SMS achieved optimal unbiasedness in heritability estimation at *S* = −0.5, yielding a heritability estimate of 0.809.

**Figure 6:**
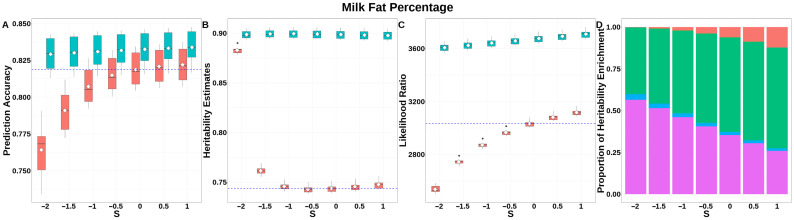
Performance of GBLUP-S (red) and GBLUP-SMS (cerulean blue) at different assumed *S*-values for fat percentage in Holstein. (**A**) Genomic prediction, (**B**) heritability estimation, (**C**) LRT statistics, and (**D**) heritability enrichment propositions across MAF bins obtained using GBLUP-SMS. In (**A**–**C**), white diamonds indicate mean values; blue dashed lines represent classic GBLUP. In (**D**), red, olive yellow, dark green, blue, and purple correspond to MAF bins 0.01–0.1, 0.1–0.2, 0.2–0.3, 0.3–0.4, and 0.4–0.5, respectively.

For FP (Figure 6, Table S2), GEBV accuracies for GBLUP-S increased monotonically with S, peaking at S = 1, while GBLUP-SMS maintained stable accuracies across all S-values. Compared to classic GBLUP, the maximum accuracy improvement reached 0.015 (GBLUP-SMS at S = 1 vs. classic GBLUP). The integrated analysis of LRT statistics, MAF-stratified enrichment ratios across varying S-values in the GBLUP-SMS model, and their corresponding chi-square statistics (Table S5) demonstrated that GBLUP-SMS achieves optimal unbiasedness in heritability estimation at S = 1, yielding a heritability estimate of 0.897.

**Figure 7:**
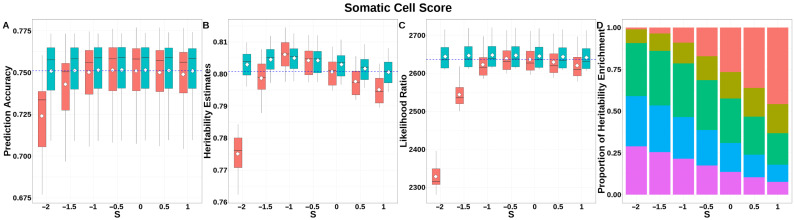
Performance of GBLUP-S (red) and GBLUP-SMS (cerulean blue) at different assumed *S*-values for somatic cell score in Holstein. (**A**) Genomic prediction, (**B**) heritability estimation, (**C**) LRT statistics, and (**D**) heritability enrichment propositions across MAF bins obtained using GBLUP-SMS. In (**A**–**C**), white diamonds indicate mean values; blue dashed lines represent classic GBLUP. In (**D**), red, olive yellow, dark green, blue, and purple correspond to MAF bins 0.01–0.1, 0.1–0.2, 0.2–0.3, 0.3–0.4, and 0.4–0.5, respectively.

For SCS (Figure 7, Table S2), both GBLUP-S and GBLUP-SMS achieved stabilized GEBV accuracies at *S* ≥ −1, with no significant differences between methods in this range. The integrated analysis of LRT statistics, MAF-stratified enrichment ratios across varying *S*-values in the GBLUP-SMS model, and their corresponding chi-square statistics (Table S6) demonstrated that GBLUP-SMS achieved optimal unbiasedness in heritability estimation at *S* = 0, yielding a heritability estimate of 0.800.

#### 3.3.2. Pig

**Figure 8:**
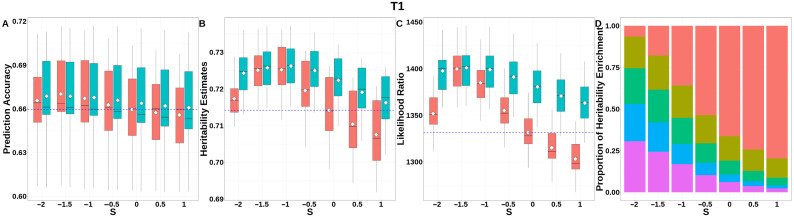
Performance of GBLUP-S (red) and GBLUP-SMS (cerulean blue) at different assumed *S*-values for T1 in pig. (**A**) Genomic prediction, (**B**) heritability estimation, (**C**) LRT statistics, and (**D**) heritability enrichment propositions across MAF bins obtained using GBLUP-SMS. In (**A**–**C**), white diamonds indicate mean values; blue dashed lines represent classic GBLUP. In (**D**), red, olive yellow, dark green, blue, and purple correspond to MAF bins 0.01–0.1, 0.1–0.2, 0.2–0.3, 0.3–0.4, and 0.4–0.5, respectively.

For T1 (Figure 8, Table S3), GBLUP-S demonstrated a prediction accuracy improvement of ~0.01 compared to classic GBLUP at *S* = 1.5. The integrated analysis of LRT statistics, MAF-stratified enrichment ratios across varying *S*-values in the GBLUP-SMS model, and their corresponding chi-square statistics (Table S7) revealed optimal unbiasedness in heritability estimation under strong negative selection (*S* = −1.5), yielding a heritability estimate of 0.725.

**Figure 9:**
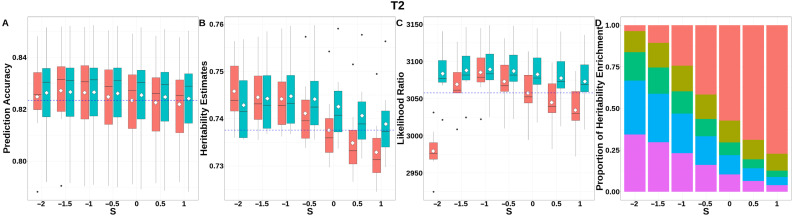
Performance of GBLUP-S (red) and GBLUP-SMS (cerulean blue) at different assumed *S*-values for T2 in pig. (**A**) Genomic prediction, (**B**) heritability estimation, (**C**) LRT statistics, and (**D**) heritability enrichment propositions across MAF bins obtained using GBLUP-SMS. In (**A**–**C**), white diamonds indicate mean values; blue dashed lines represent classic GBLUP. In (**D**), red, olive yellow, dark green, blue, and purple correspond to MAF bins 0.01–0.1, 0.1–0.2, 0.2–0.3, 0.3–0.4, and 0.4–0.5, respectively.

For T2 (Figure 9, Table S3), GEBV prediction accuracies for both methods were indistinguishable across all *S*-values. The integrated analysis of LRT statistics, MAF-stratified enrichment ratios across varying *S*-values in the GBLUP-SMS model, and their corresponding chi-square statistics (Table S8) revealed that optimal unbiasedness in heritability estimation was achieved under negative selection (*S* = −1), yielding a heritability estimate of 0.744.

**Figure 10:**
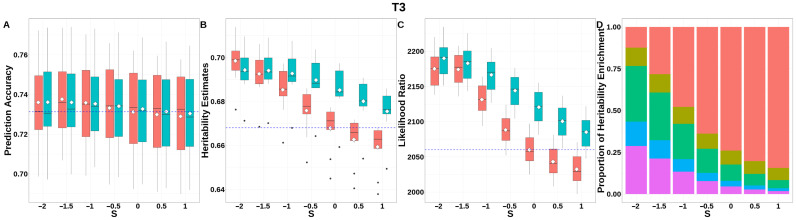
Performance of GBLUP-S (red) and GBLUP-SMS (cerulean blue) at different assumed *S*-values for T3 in pig. (**A**) Genomic prediction, (**B**) heritability estimation, (**C**) LRT statistics, and (**D**) heritability enrichment propositions across MAF bins obtained using GBLUP-SMS. In (**A**–**C**), white diamonds indicate mean values; blue dashed lines represent classic GBLUP. In (**D**), red, olive yellow, dark green, blue, and purple correspond to MAF bins 0.01–0.1, 0.1–0.2, 0.2–0.3, 0.3–0.4, and 0.4–0.5, respectively.

For T3 (Figure 10, Table S3), GEBV prediction accuracies for both methods remained indistinguishable across all *S*-values. The integrated analysis of LRT statistics, MAF-stratified enrichment ratios across varying *S*-values in the GBLUP-SMS model, and their corresponding chi-square statistics (Table S9) revealed that optimal unbiasedness in heritability estimation was achieved under strong negative selection (*S* = −2), yielding a heritability estimate of 0.694.

## 4. Discussion

### 4.1. Performance of Models in Simulation Analysis

The GRM constructed with parameter *S* = 0 in the GBLUP-S framework, equivalent to the classic GBLUP [10], remains the most widely applied approach in current animal and plant breeding, which assumes equal variance across all SNP effects while disregarding the impact of natural or artificial selection on genetic architecture, i.e., ignoring the relationship between MAF and marker effects. Notably, the GCTA implements the GBLUP-S model under *S* = −1, which postulates a strong negative correlation between MAF and marker effects, along with equal heritability contributions from each SNP [14].

Our study demonstrated that integrating selection-adjusted genetic structure into GRM construction through distinct genotype coding standardization strategies greatly influences the performance of heritability estimation and genomic prediction. This finding aligns with simulation results derived from human data [28]. Given the heterogeneity in genetic architectures across traits due to divergent selection pressures in domesticated species, the true selection signatures likely vary. Incorporating the relationship between marker effects and MAF may thus refine heritability estimation and genetic prediction models for livestock. Specifically, our results indicate that when the *S* value used in models matches or approximates the true selection signature, selection-adjust methods achieve higher heritability estimates while maintaining unbiasedness and genomic prediction accuracy. For empirical datasets, cross-validation within reference populations could provide preliminary inference of plausible ranges for *S*.

In human genetics, Bayesian methods such as BayesS [29] employ Markov Chain Monte Carlo (MCMC) algorithms to estimate trait-specific selection signatures. However, BayesS exhibits limited efficacy for traits with small phenotypic datasets. Consequently, its adaptation to large-scale breeding populations merits exploration to enhance heritability estimation and genetic evaluation performance [15].

For human heritability analyses, stringent quality control typically removes closely related individuals (e.g., excluding pairs with GRM values > 0.025) [19,30] to mitigate inflation from shared environmental confounders, thereby ensuring narrow-sense heritability estimates [31,32]. In contrast, limited population sizes in animal and plant breeding often preclude the establishment of unrelated population for reliable heritability estimation [19,33]. Our simulations revealed that GBLUP-S or GBLUP-SMS heritability estimates for traits with true *h*^2^ = 0.1 were systematically larger than the true values, partly attributable to unaccounted shared environmental variance, suggesting that GBLUP-S or GBLUP-SMS estimates in breeding populations conflate SNP-associated variance with non-genetic factors, deviating from narrow-sense heritability definitions [34].

Notably, GBLUP-SMS demonstrated superior stability in heritability estimation and genomic prediction compared to GBLUP-S, with minimal performance fluctuations across varying *S-*values. This robustness implies that modeling distinct MAF distributions across genomic regions enhances adaptability to diverse genetic architectures [15].

### 4.2. Performance of Models on Real Data

Our findings indicate that the selection signature parameter S greatly affects heritability estimates and GEBV accuracy. Incorporating selection signatures (e.g., marker effect–MAF relationships) into genomic models may enhance heritability estimation unbiasedness through causal quantitative trait loci (QTL)-linked SNPs identification, with modest gains in genomic prediction.

For the GBLUP-S method, selecting an appropriate *S* value aligned with the trait’s genetic architecture is critical to achieving optimal heritability estimates or GEBV accuracy, consistent with its theoretical assumptions [26]. In contrast, GBLUP-SMS exhibits reduced sensitivity to *S* variations, though its performance remains partially influenced by parameter selection. For instance, in pig T3, the stratification of SNPs by MAF bins in GBLUP-SMS, which constructs separate GRMs for distinct MAF bins with independent distributional assumptions, may mitigate discrepancies between genetic architectures and model hypotheses (selection signature), particularly for traits governed by large- or moderate-effect genes.

In real phenotypic data, similar to simulated data, the presence of closely related individuals within populations can lead to relatively larger heritability estimates. However, unlike simulated scenarios where QTL positions are predefined, the unknown genomic locations of causal QTL in empirical data exacerbate the issue of ‘missing’ or ‘hidden’ heritability. Specifically, causal variants with true effects may fail to be captured due to either (1) incomplete linkage disequilibrium (LD) between QTL and genotyped SNPs on commercial panels, or (2) QTL with minor allele frequencies (MAF) too low (<0.01) to establish sufficient LD with nearby markers, collectively contributing to an underestimation of heritability [13].

## 5. Conclusions

Under the GBLUP model framework, incorporating the relationship between MAF and marker effect sizes caused by natural or artificial selection when constructing the GRM can improve the performance of heritability estimation and genomic prediction. Additionally, the GBLUP-SMS method demonstrates greater stability compared to GBLUP-S, as it does not rely on specifying *S* parameters in the model. The chi-square test serves as an effective approach to validate the performance of heritability estimation and genomic prediction.

## Data Availability

The data (Holstein and pig) are publicly available from G3 (https://pmc.ncbi.nlm.nih.gov/articles/instance/4390577/bin/supp_5_4_615__index.html (accessed on 1 February 2024)) and (https://pmc.ncbi.nlm.nih.gov/articles/instance/3337471/bin/supp_2_4_429__index.html (accessed on 1 February 2024)).

## Supplementary Materials

The following supporting information can be downloaded at: https://www.mdpi.com/article/10.3390/ani15101383/s1, Table S1: Performance of GBLUP-S and GBLUP-SMS for simulated data in terms of genomic prediction and heritability estimation. Table S2: Performance of GBLUP-S and GBLUP-SMS for Holstein in terms of genomic prediction and heritability estimation. Table S3: Performance of GBLUP-S and GBLUP-SMS for a pig in terms of genomic prediction and heritability estimation. Table S4: Heritability enrichment ratio and chi-square values within each MAF bin of milk yield of Holstein. Table S5: Heritability enrichment ratio and chi-square values within each MAF bin of milk fat percentage of Holstein. Table S6: Heritability enrichment ratio and chi-square values within each MAF bin of somatic cell score of Holstein. Table S7: Heritability enrichment ratio and chi-square values within each MAF bin of T1 of pig. Table S8: Heritability enrichment ratio and chi-square values within each MAF bin of T2 of pig. Table S9: Heritability enrichment ratio and chi-square values within each MAF bin of T3 of pig.
