# Supplementary material for: Impact of Selection Signature on Genomic Prediction and Heritability Estimation in Livestock
Source: Animals (Basel). 2025 May 10;15(10):1383. doi: 10.3390/ani15101383 (PMC12108319; doi:10.3390/ani15101383)
Supplement: Supplementary file 1 [file animals-15-01383-s001.zip › animals-3587334 - Supplementart.pdf]

Table S1. Performance of GBLUP-S and GBLUP-SMS for simulated data in terms of genetic evaluation and heritability estimation, mean±SD.

True S (Selection Signature)	S Applied in model	Prediction Accuracy		Heritability Estimates	
		GBLUP-S	GBLUP-SMS	GBLUP-S	GBLUP-SMS
-2	-2	0.5947±0.0336	0.5895±0.0363	0.1127±0.0149	0.1157±0.0151
	-1.5	0.5883±0.0285	0.5882±0.0342	0.1105±0.0168	0.1164±0.0158
	-1	0.5769±0.0267	0.585±0.0318	0.1046±0.018	0.1158±0.0158
	-0.5	0.5687±0.0262	0.5795±0.0294	0.1003±0.0186	0.1152±0.0146
	0	0.5638±0.026	0.5761±0.0297	0.0978±0.0189	0.1113±0.0168
	0.5	0.5608±0.0258	0.5734±0.0294	0.0962±0.019	0.1096±0.0171
	1	0.5587±0.0257	0.5711±0.0293	0.0951±0.0191	0.1086±0.0171
-1.5	-2	0.5863±0.0339	0.5875±0.0347	0.1156±0.0136	0.1221±0.0173
	-1.5	0.5929±0.0296	0.5872±0.0341	0.117±0.0126	0.1228±0.017
	-1	0.5893±0.0284	0.5857±0.0334	0.1127±0.011	0.123±0.0175
	-0.5	0.5849±0.0288	0.5835±0.0327	0.1088±0.0099	0.1218±0.0177
	0	0.5817±0.0293	0.5817±0.0322	0.1063±0.0093	0.1189±0.0165
	0.5	0.5795±0.0298	0.5802±0.0319	0.1047±0.0089	0.117±0.0162
	1	0.5777±0.0302	0.579±0.0318	0.1035±0.0086	0.116±0.0157
-1	-2	0.5714±0.0396	0.5715±0.0441	0.1037±0.0075	0.1147±0.0152
	-1.5	0.582±0.0405	0.5725±0.0445	0.1066±0.0052	0.1146±0.0146
	-1	0.5814±0.0414	0.5736±0.045	0.1044±0.0049	0.1143±0.0136
	-0.5	0.5791±0.0419	0.5739±0.045	0.1019±0.005	0.1134±0.0132
	0	0.5772±0.0422	0.5736±0.0447	0.1001±0.0051	0.1136±0.0135
	0.5	0.5758±0.0423	0.5731±0.0443	0.0989±0.0051	0.1133±0.0137
	1	0.5748±0.0423	0.5727±0.044	0.098±0.0052	0.1126±0.0141
-0.5	-2	0.5453±0.0343	0.571±0.0316	0.1037±0.0132	0.1081±0.0121
	-1.5	0.5687±0.0327	0.5711±0.0316	0.1075±0.0129	0.1094±0.0132
	-1	0.5754±0.0323	0.5715±0.0317	0.1057±0.0122	0.1084±0.0129
	-0.5	0.5767±0.0321	0.5718±0.0317	0.1036±0.0118	0.1082±0.0134
	0	0.5766±0.0321	0.5717±0.0316	0.1021±0.0115	0.1082±0.0149
	0.5	0.5761±0.032	0.5712±0.0315	0.1011±0.0114	0.1073±0.0136
	1	0.5755±0.032	0.5705±0.0315	0.1004±0.0113	0.1077±0.0135
0	-2	0.554±0.0473	0.5796±0.0513	0.0973±0.0069	0.1096±0.0173
	-1.5	0.5785±0.0487	0.5801±0.052	0.1004±0.0065	0.1094±0.0187
	-1	0.5865±0.0488	0.5801±0.0526	0.0983±0.0067	0.1094±0.0189
	-0.5	0.5889±0.0488	0.58±0.0525	0.096±0.007	0.1094±0.0205
	0	0.5895±0.0488	0.5801±0.0522	0.0945±0.0071	0.1082±0.0202
	0.5	0.5895±0.049	0.5801±0.0518	0.0934±0.0072	0.1069±0.0187
	1	0.5893±0.0491	0.5801±0.0514	0.0926±0.0072	0.1057±0.0176
0.5	-2	0.5504±0.0357	0.5758±0.0346	0.1062±0.0178	0.1258±0.026
	-1.5	0.5741±0.0339	0.5746±0.0355	0.1108±0.019	0.125±0.0273

	-1	0.5819±0.035	0.5743±0.0358	0.1093±0.0188	0.1241±0.0272
	-0.5	0.584±0.0363	0.5751±0.0354	0.1072±0.0183	0.1217±0.0261
	0	0.5844±0.0373	0.575±0.0351	0.1056±0.0178	0.1222±0.0259
	0.5	0.5843±0.0379	0.5753±0.0349	0.1045±0.0174	0.122±0.0242
	1	0.584±0.0384	0.5755±0.0349	0.1036±0.0172	0.1213±0.0238
1	-2	0.543±0.0513	0.571±0.0471	0.1047±0.0162	0.1194±0.016
	-1.5	0.5689±0.0514	0.5706±0.0473	0.1098±0.0143	0.1201±0.0156
	-1	0.5785±0.0517	0.5702±0.0476	0.1086±0.0125	0.1209±0.0148
	-0.5	0.5818±0.0518	0.57±0.0474	0.1066±0.0115	0.1203±0.0158
	0	0.5831±0.0518	0.5702±0.047	0.1051±0.011	0.1198±0.0168
	0.5	0.5835±0.0518	0.5708±0.0468	0.1041±0.0107	0.1201±0.0166
	1	0.5835±0.0518	0.5711±0.0467	0.1033±0.0105	0.1199±0.0171

Table S2. Performance of GBLUP-S and GBLUP-SMS for Hostein in terms of genetic evaluation and heritability estimation, mean±SD.

True S (Selection Signature)	S Applied in model	Prediction Accuracy		Heritability Estimates	
		GBLUP-S	GBLUP-SMS	GBLUP-S	GBLUP-SMS
MY	-2	0.7472±0.0208	0.7769±0.0228	0.7617±0.0063	0.8123±0.017
	-1.5	0.7668±0.0203	0.7788±0.0202	0.7957±0.0069	0.8082±0.0076
	-1	0.776±0.0201	0.7792±0.02	0.8108±0.0072	0.809±0.0076
	-0.5	0.7792±0.0202	0.7796±0.0199	0.8139±0.0072	0.8093±0.0076
	0	0.78±0.0204	0.7799±0.0199	0.8129±0.0072	0.8092±0.0076
	0.5	0.78±0.0205	0.7801±0.0199	0.8111±0.0071	0.8089±0.0076
	1	0.7798±0.0206	0.7803±0.02	0.8091±0.0071	0.8086±0.0076
FP	-2	0.7641±0.0173	0.8292±0.0114	0.8828±0.0036	0.8986±0.0041
	-1.5	0.791±0.0146	0.8301±0.0114	0.7615±0.0039	0.8993±0.0042
	-1	0.8073±0.013	0.831±0.0115	0.7456±0.0036	0.8995±0.0043
	-0.5	0.815±0.0121	0.8318±0.0114	0.7428±0.0037	0.8992±0.0044
	0	0.8188±0.0118	0.8325±0.0114	0.7437±0.0039	0.8987±0.0045
	0.5	0.8209±0.0116	0.8332±0.0114	0.7456±0.0041	0.8981±0.0045
	1	0.822±0.0114	0.8339±0.0113	0.7477±0.0043	0.8976±0.0044
SCS	-2	0.724±0.0247	0.7509±0.0208	0.7751±0.0067	0.8029±0.0045
	-1.5	0.743±0.0231	0.7513±0.0209	0.7987±0.006	0.8045±0.0046
	-1	0.7502±0.0218	0.7515±0.0208	0.8061±0.0053	0.805±0.0045
	-0.5	0.7515±0.0213	0.7516±0.0208	0.8042±0.0048	0.8042±0.0045
	0	0.751±0.0214	0.7515±0.0207	0.8007±0.0046	0.8029±0.0044
	0.5	0.75±0.0215	0.7513±0.0206	0.7977±0.0046	0.8017±0.0044
	1	0.749±0.0217	0.7511±0.0206	0.7951±0.0046	0.8006±0.0044

Table S3. Performance of GBLUP-S and GBLUP-SMS for pig in terms of genetic evaluation and heritability estimation, mean±SD.

True S (Selection Signature)	S Applied in model	Prediction Accuracy		Heritability Estimates	
		GBLUP-S	GBLUP-SMS	GBLUP-S	GBLUP-SMS
T1	-2	0.6657±0.0313	0.6686±0.0311	0.7173±0.0055	0.7243±0.0069
	-1.5	0.6701±0.0316	0.6686±0.0317	0.7251±0.0064	0.7258±0.0067
	-1	0.6671±0.0315	0.6676±0.0322	0.7252±0.0078	0.7262±0.0067
	-0.5	0.6627±0.0306	0.6659±0.0325	0.7196±0.009	0.725±0.0069
	0	0.6596±0.0297	0.6637±0.0325	0.7142±0.0097	0.7223±0.0074
	0.5	0.6574±0.0291	0.6619±0.0323	0.7104±0.0101	0.7191±0.0078
	1	0.6558±0.0288	0.6607±0.0319	0.7076±0.0103	0.7163±0.0082
T2	-2	0.8248±0.0162	0.8263±0.0163	0.7458±0.0068	0.7429±0.0074
	-1.5	0.8272±0.0167	0.8267±0.0166	0.7445±0.007	0.7442±0.0074
	-1	0.8264±0.0171	0.8266±0.0168	0.7442±0.0071	0.7448±0.0073
	-0.5	0.8248±0.0171	0.8261±0.0171	0.7411±0.0072	0.7441±0.0073
	0	0.8234±0.017	0.8254±0.0173	0.7376±0.0073	0.7425±0.0074
	0.5	0.8225±0.0169	0.8247±0.0174	0.7349±0.0073	0.7406±0.0075
	1	0.8219±0.0168	0.8241±0.0174	0.7329±0.0073	0.7389±0.0076
T3	-2	0.7361±0.0235	0.7361±0.0238	0.6986±0.0108	0.6944±0.0111
	-1.5	0.7377±0.0223	0.7361±0.0238	0.6926±0.0109	0.6941±0.0112
	-1	0.7358±0.0227	0.7354±0.0238	0.6854±0.0107	0.6928±0.0113
	-0.5	0.7333±0.0235	0.7341±0.0238	0.6759±0.010	0.6898±0.0112
	0	0.7313±0.0241	0.7327±0.024	0.668±0.0106	0.6852±0.011
	0.5	0.7299±0.0243	0.7315±0.0241	0.6627±0.0106	0.6801±0.0107
	1	0.729±0.0244	0.7304±0.0242	0.6593±0.0106	0.6755±0.0104

Table S4. Heritability enrichment ratio and chi-square values within each MAF bin of milk yield of Holstein.

**MAF Bin**	**Selection signature**						
	**-2**	**-1.5**	**-1**	**-0.5**	**0**	**0.5**	**1**
0.01<=MAF<0.1	0.02	0.037	0.1	0.201	0.322	0.445	0.56
0.1<=MAF<0.2	0.067	0.089	0.101	0.111	0.119	0.121	0.117
0.2<=MAF<0.3	0.297	0.319	0.325	0.309	0.274	0.229	0.183
0.3<=MAF<0.4	0.139	0.129	0.111	0.09	0.07	0.052	0.037
0.4<=MAF<0.5	0.477	0.427	0.364	0.289	0.216	0.152	0.103
χ^2	0.517	0.382	0.247	0.158	0.206	0.425	0.785

Table S5. Heritability enrichment ratio and chi-square values within each MAF bin of milk fat percentage of Holstein.

**MAF Bin**	**Selection signature**						
	**-2**	**-1.5**	**-1**	**-0.5**	**0**	**0.5**	**1**
0.01<=MAF<0.1	0.002	0.008	0.021	0.039	0.061	0.088	0.123
0.1<=MAF<0.2	0.001	0.001	0.001	0.001	0.001	0	0
0.2<=MAF<0.3	0.399	0.449	0.495	0.534	0.566	0.59	0.603
0.3<=MAF<0.4	0.034	0.028	0.023	0.02	0.019	0.017	0.016
0.4<=MAF<0.5	0.564	0.514	0.46	0.406	0.353	0.304	0.258
χ^2	0.991	0.978	0.954	0.923	0.891	0.86	0.827

Table S6. Heritability enrichment ratio and chi-square values within each MAF bin of somatic cell score of Holstein.

**MAF Bin**	**Selection signature**						
	**-2**	**-1.5**	**-1**	**-0.5**	**0**	**0.5**	**1**
0.01<=MAF<0.1	0.011	0.036	0.091	0.172	0.266	0.362	0.458
0.1<=MAF<0.2	0.083	0.104	0.124	0.143	0.16	0.171	0.174
0.2<=MAF<0.3	0.318	0.327	0.321	0.298	0.266	0.228	0.189
0.3<=MAF<0.4	0.301	0.28	0.25	0.212	0.173	0.136	0.103
0.4<=MAF<0.5	0.288	0.254	0.215	0.174	0.136	0.103	0.076
χ^2	0.455	0.32	0.175	0.068	0.054	0.148	0.346

Table S7. Heritability enrichment ratio and chi-square values within each MAF bin of T1 of pig.

**MAF Bin**	Selection signature						
	-2	-1.5	-1	-0.5	0	0.5	1
0.01<=MAF<0.1	0.066	0.179	0.359	0.537	0.663	0.743	0.797
0.1<=MAF<0.2	0.189	0.205	0.195	0.17	0.147	0.13	0.115
0.2<=MAF<0.3	0.216	0.196	0.158	0.116	0.084	0.062	0.046
0.3<=MAF<0.4	0.223	0.177	0.121	0.074	0.045	0.028	0.019
0.4<=MAF<0.5	0.306	0.243	0.167	0.103	0.061	0.037	0.023
χ^2	0.092	0.003	0.127	0.585	1.128	1.574	1.928

Table S8. Heritability enrichment ratio and chi-square values within each MAF bin of T2 of pig.

**MAF Bin**	Selection signature						
	-2	-1.5	-1	-0.5	0	0.5	1
0.01<=MAF<0.1	0.035	0.107	0.244	0.417	0.572	0.689	0.772
0.1<=MAF<0.2	0.127	0.147	0.153	0.146	0.132	0.117	0.102
0.2<=MAF<0.3	0.17	0.158	0.134	0.104	0.076	0.054	0.038
0.3<=MAF<0.4	0.324	0.292	0.237	0.173	0.117	0.077	0.049
0.4<=MAF<0.5	0.344	0.296	0.231	0.161	0.103	0.064	0.039
χ^2	0.241	0.099	0.053	0.294	0.787	1.334	1.831

Table S9. Heritability enrichment ratio and chi-square values within each MAF bin of T3 of pig.

**MAF Bin**	**Selection signature**						
	**-2**	**-1.5**	**-1**	**-0.5**	**0**	**0.5**	**1**
0.01<=MAF<0.1	0.124	0.283	0.479	0.638	0.74	0.803	0.844
0.1<=MAF<0.2	0.11	0.11	0.101	0.091	0.083	0.077	0.072
0.2<=MAF<0.3	0.333	0.286	0.212	0.145	0.099	0.069	0.05
0.3<=MAF<0.4	0.145	0.11	0.075	0.049	0.033	0.023	0.017
0.4<=MAF<0.5	0.288	0.212	0.133	0.077	0.045	0.027	0.017
χ^2	0.191	0.198	0.586	1.2	1.733	2.118	2.404
